# Healthcare workers' readiness for artificial intelligence and organizational change: a quantitative study in a university hospital

**DOI:** 10.1186/s12913-025-12846-y

**Published:** 2025-06-08

**Authors:** Hafize Boyacı, Selma Söyük

**Affiliations:** 1 Fikret Biyal Central Research Laboratory, Cerrahpasa Medical Faculty, Istanbul University-Cerrahpasa, Istanbul, Türkiye; 2 Department of Health Management, Faculty of Health Sciences, Istanbul University-Cerrahpasa, Istanbul, Türkiye

**Keywords:** Artificial intelligence, Organizational change, Health institution, Healthcare, Structural equation model

## Abstract

**Objective:**

The aim of the study is to measure the readiness levels of medical artificial intelligence and the perception of openness to organizational change of healthcare professionals working in a university hospital in Istanbul. Additionally, the study seeks to identify the relationships between medical AI readiness and perceptions of organizational change openness, as well as to examine differences based on demographic variables.

**Method:**

The research was conducted with 195 healthcare workers. The research is a cross-sectional descriptive quantitative research. The construct validity of the scales was checked using statistical analysis.

**Result:**

As a result of the research, it was determined that healthcare workers’ are prepared for the use of medical artificial intelligence in healthcare institutions and perceive organizational change positively. A significant but low-level positive relationship was found between healthcare workers’ level of readiness for medical artificial intelligence and their perception of openness to organizational change. The level of readiness for medical artificial intelligence among healthcare workers’ was found to be high among males, doctors and internal sciences, while the perception of openness to organizational change was found to be high among postgraduate/doctoral graduates, surgical sciences, nurses.

**Conclusion:**

The study determined that healthcare workers’ are ready to use medical artificial intelligence and perceive organizational change positively. The study contributes to the formation of the institution's healthcare policies and practices and to the development, well-being and change of healthcare workers’. It is recommended that employees be made aware of the benefits of using artificial intelligence in healthcare institutions and that necessary training activities be planned.

## Background

Technology offers new solutions to improve the quality of healthcare and facilitate access [1]. The application of AI in healthcare requires being prepared for the opportunities that technological developments will offer. Healthcare institutions need to be prepared for AI applications for sustainable quality healthcare service delivery. It is thought that AI can help in diagnosis, treatment, reducing malpractice risks and treating more patients in healthcare applications [2]. The use of AI in healthcare institutions will undoubtedly lead to change and contribute to the development of the institution. Healthcare institutions will also need to be redesigned according to the technological opportunities of AI [3]. In this process, healthcare workers will also need to be open to new ideas and perspectives, adapt to change and be flexible in the face of changes [4].

### Artificial intelligence

The rapidly increasing use of AI in the world has led to the need to explain exactly what the concept of AI is. The term "artificial intelligence" was first used in 1956 by John McCarthy and his team in a summer project proposal at Dartmouth University [5]. According to another definition, it is explained as "the theory and development of computer systems capable of performing tasks that require human intelligence (such as decision making, visual perception, speech recognition, and translation between languages)" [6]. The Australian Council of Learned Academies defines AI as "a collection of technologies that are associated with each other to perform tasks and solve problems that require human thinking" [7]. The definition of AI by the OECD is as follows; "a machine-based system that can make predictions, recommendations or decisions affecting real or virtual environments for a specific set of human-defined goals. AI systems are designed to operate with varying degrees of independence" [8]. In the age of technology, automation and AI are defined as a series of programs that are compatible with computers and will perform many functions together [9]. Despite its complex structure, its use is rapidly spreading. There are many decision support systems developed with AI technology [10]. AI is the system used to solve complex tasks and processes that arise by imparting human thinking skills to machines with the help of computers [11].

In this context, AI technology is also adapting very quickly in the health sector. The benefits of using AI in the health sector are quite high. AI is used in the field of health for medical purposes, imaging, diagnosis, treatment applications, as well as management systems, software, and documentation applications [12]. AI applications are used as an important tool in planning activities and documentation in health systems with complex organizational structures. It helps to increase the quality of service delivery, provide a certain standard and organize hospital management applications [13, 14]. In order to establish standards in healthcare and increase service efficiency, it is imperative to integrate contemporary applications such as artificial intelligence, machine learning, and the Internet of Things into hospitals. Modern applications that focus on problem solving, are useful and provide energy saving offer great benefits in the field of health [15]. In terms of hospital management systems, it is seen that the use of AI is necessary for the management of processes, resource allocation and patient information system management to create standards [13, 16]. Another area of use of AI is monitoring patient test results. Applications that are monitored by physicians with the help of artificial intelligence-supported wearable technological devices are used [12]. AI also has benefits for healthcare workers. Studies have shown that it has positive effects on physicians' performance. It includes systems that can analyze physiological problems experienced by patients and provide appropriate intervention techniques [17]. It is stated that artificial intelligence-supported medical imaging provides more accurate and faster diagnoses in MRI scans [18].

There may be disadvantages as well as advantages of AI. In the long term, it may replace humans and there may be job loss [3]. Abdullah and Fakieh [19] believe that most healthcare workers will lose their jobs due to AI. Another concern about AI is that healthcare workers are worried that robots that lack empathy and emotion will perform surgeries, treatments and examinations [20–23].

Insufficient knowledge about the capabilities of various AI technologies among healthcare workers and organizations is also a disadvantage [24]. Discussions about the ethical aspects of AI continue. Legal and ethical regulations are required for safe AI design, considering the danger of AI developing superior to humans in the future [25]. More research is needed to ensure ethical design of AI. There are also views that argue that AI should be used with caution in healthcare services, despite its usefulness in healthcare systems and the process of improving health [26].

### Openness toward organizational change

Adapting to technological developments and managing the process is a targeted situation in terms of the development of organizations [27]. While defining change in the literature, Kurt Lewin (act. [28]) stated that change is "the transition from a current situation to a new situation and is realized through changes in the psychological forces in the living space". Change enables the institution to gain a new quality [29]. In change management, the adoption of the designed change movement by employees ensures the achievement of business goals [30]. Evaluating new methods and methods and using previously developed change strategies can increase the chance of success [31]. The success or failure of the change process varies between supporting or resisting the change [28, 32]. Preventing resistance in organizations and trying to reduce conflict during change will increase the success of the change [33]. The reason for resistance to change is the lack of information about why the change is being made and the lack of knowledge about what the results will be [30]. The success of change is achieved by ensuring internal communication [34]. The perspective of managers and employees on change differs [35]. Technological developments cause employees to think that they will encounter inadequacy and unemployment problems [36]. On the contrary, employees who are open to change make positive contributions to the organization by adapting to a certain plan emotionally and cognitively. The principle of employees' integration with their purpose lies at the basis of achieving the organization's goal. It is necessary to keep up with the rapidly developing technology and innovations in treatment practices, especially in health institutions [37]. Health institutions are the institutions that use technology the most intensively and consume it the fastest after space studies. In these institutions, the adaptation of personnel to technological change and all changes that may occur is important and this affects all organizational results [38]. In addition, ensuring continuous training of health personnel necessitates the use of modern management techniques. Openness to change is the formation of the desire to adapt to and accept change. Miller et al. [39] defined openness to change as a combination of the willingness to contribute to change and the positive impact on the possible results of change. Openness to change at the organizational level is an important factor that reduces resistance and increases the chance of success of change [40]. In the change process carried out with the participation of employees, their understanding of the change that is an organizational need affects the attitude of employees. It is important for managers to determine the openness of employees to change and its impact [41]. Organizations can increase their openness to change by sharing information with employees, creating an environment of trust between them and seeing them as a part of the change [42].

Artificial intelligence has rapidly penetrated all sectors globally, and its support is now utilized in numerous fields. It is anticipated that AI will also be swiftly integrated into the healthcare sector; therefore, it is essential to assess the readiness of healthcare professionals for this transition. The healthcare sector is among the most intensive users of technology—an imperative rather than a choice. Otherwise, there is a significant risk of falling behind in diagnosis and treatment processes.

As with any innovation, the introduction of AI leads to organizational change, and the extent to which employees can quickly adapt to these changes is believed to directly influence the success of such transitions. In cases where resistance to change exists, it becomes crucial to enhance the acceptance of AI applications before their widespread implementation in healthcare. With this study, we aim to draw the attention of both healthcare workers and administrators to this issue.

Measuring and evaluating employees' perceptions of organizational change is important for healthcare institution management practices. It is important to question healthcare professionals' openness to change in the organization and to be prepared for possible resistance to innovations to be implemented in the healthcare institution in the future. It is thought that the results of the study will contribute to institution managers and the institution from a managerial perspective and guide future studies. In order to determine the multifaceted effect of AI on organizational change, we think that determining healthcare workers' AI readiness level and perception of openness to organizational change will contribute to the successful integration of AI into healthcare institutions in the future. At the same time, we think that the relationship between openness to change and AI will be a step for healthcare managers to plan their management and applications. The study aims to determine the medical artificial intelligence readiness and organizational change perception of healthcare professionals working in healthcare institutions. It also aims to reveal the differences in variables according to demographic characteristics. According to the method, it is aimed to measure the relationship between healthcare professionals' readiness for medical artificial intelligence in healthcare institutions and healthcare professionals' perception of openness to organizational change.

## Materials and methods

The research is a cross-sectional descriptive quantitative research. The study was conducted by applying a survey form to healthcare workers at a university hospital in Istanbul. In the study, the relationship between healthcare workers’ medical AI readiness and their perception of openness to organizational change was determined using Pearson correlation coefficient and Structural Equation Modeling (SEM).

### Data collection tool

In the research, validity and reliability studies were conducted and a questionnaire form was created using scales that were evaluated to be highly reliable. The questionnaire comprised three sections. The first section comprises a demographic information form designed to measure the heterogeneity of participants based on socio-demographic characteristics, including gender, age, education, work time in the profession and Institution and department. And descriptive information containing general information about AI is included.

#### Medical Artificial Intelligence Readiness Scale for Medical Students (MAIRS-MS)

In the second part of the questionnaire form, the “MAIRS-MS” scale consisting of 22 statements developed by Karaca et al. [43] was used. The “MAIRS-MS” Scale, which was developed to determine the perceived readiness of medical students for medical AI, consists of 4 sub-factors as Cognitive Factor, Ability Factor, Vision Factor and Ethical Factor. The 22 statements of the 4-factor structure of the Medical AI Readiness Scale have min: 22-max: 110 points. The answers to the Likert-type 5-point scale are 1- Strongly disagree, 2- Disagree 3- Neutral 4- Agree, 5- Strongly agree. The validity and reliability studies of the scale were conducted by Karaca et al. [43]. The Cronbach alpha reliability coefficient of the scale is 0.87. The scale was preferred for use in the study because it has high validity and reliability. Although the scale was developed to determine the readiness of medical students to AI, it was used in the study to determine the readiness of healthcare workers (physicians, nurses, etc.) working in a university hospital to the use of AI applications in healthcare institutions. Permission for the use of the scale was received from Karaca et al. via e-mail on 10.09.2024.

#### Openness toward organizational change scale (OTOC)

In the third part of the survey, in order to determine the attitudes and behaviors of healthcare workers against the changes caused by technological developments in healthcare institutions, the “OTOC” scale developed by Çalışkan A. [44] was applied to a working group consisting of education, industry and healthcare sector employees. In the study, the OTOC scale was used to healthcare workers. The scale developed by Çalışkan [44] is one-dimensional and consists of 6 items.

The validity and reliability studies of the “OTOC” scale were conducted and the criterion-dependent validity of the scale was provided with the leader support scale. Because of the healthcare sector application, the scale with a reliability coefficient of 0.921 was preferred for use in the research. The scale was developed to measure the positive effects and results of change in organizations through the phenomenon of openness to change. The scale was developed to determine the tendency of individuals working in all businesses in Turkey to be open toward organizational change. In the study, it was used to determine the perception of openness to change of employees towards AI applications in healthcare workers. Permission to use the scale was received from Çalışkan via e-mail on 10.09.2024. The 5-point Likert-type scale was graded as 1-Strongly Disagree 2-Disagree 3-Undecided 4-Agree 5-Strongly Agree.

The aim of the study is to evaluate the attitudes of healthcare workers who provide positive impact and support for change and its results in organizations, to determine and model the relationship between healthcare workers’ perception of openness to change and their readiness for artificial intelligence.

### Population and sample

The research was conducted with healthcare workers (doctors, nurses, health technicians, medical secretaries, etc.) working in a university hospital in Istanbul, Turkey. The research universe consists of 3979 healthcare workers. Convenience sampling was preferred as it is considered to best represent the population and is the easiest, fastest, and most economical way to collect data from the main population [45]. Additionally, the high cost and time required to reach the entire population also influenced the choice of this method. If the perception, attitude, etc. of the participants on a specific subject is evaluated with survey questions, the sample size should be at least 2–10 times the number of items in order to evaluate the suitability of the sample for statistical analysis [46]. It is generally accepted that a 1:5 sample will be used. In the study where quantitative research method was used, data was collected by survey method.

The survey form consists of 37 statements. The required sample size for the study was calculated as 5 times the scale expression and the minimum sample size was 185 people. Data in the study were obtained using the convenience sampling method between December 1–30, 2024. The convenience sampling method was selected in the study because it is easy to access, fast and economical. It is the most widely used sampling strategy [47]. It is explained in the limitation section that the convenience sampling method will pose a problem in generalizing the study. It is stated that only a single health institution was considered since the AI readiness level of healthcare workers was determined in the study.

In the study, 203 healthcare workers were reached. Online and manual survey methods were applied as primary data collection methods. The surveys with incomplete and incorrect fillings were removed and the study was conducted on 195 people. The sample of the study consists of doctors, nurses, health technicians and other healthcare workers. By comparing the mean scores obtained from the medical AI readiness and openness to organizational change scale with demographic variables, healthcare workers' levels of readiness for AI and their perceptions of openness to organizational change were determined.

### Ethical aspect of the research

The research was conducted with the ethics committee approval numbered E-74555795–050.04–1156423/2024/219 and institutional permission numbered E-50200903–903.99–1 136603 from the institution where the research was conducted, and the necessary consent forms for the survey study were obtained. This study was performed in accordance with the guidelines of the Declaration of Helsinki and approved. Participants answered the survey voluntarily.

### Statistical analysis

IBM SPSS software package and Lisrel Estimated 8.8 programs were used for statistical analyses. The descriptive characteristics (age, gender, education level, working time in the profession, AI usage status) and distributions of all groups were determined. MAIRS-MS scale; item analysis, mean, percentage, frequency values, Kaiser–Meyer–Olkin (KMO) and Bartlett values, mean, standard deviation, multiple correlation values were determined. The construct validity of the scale was checked using EFA and CFA. “OTOC” Scale: item analysis, mean, percentage and frequency, KMO and Bartlett values, mean, standard deviation, multiple correlation values were determined. The construct validity of the scale was checked using EFA and CFA. The data were tested for normality distribution with skewness, kurtosis, histogram and normal distribution graph test. Parametric methods were selected for data showing normal distribution and intra-group statistical comparisons were made. In order to test the relationship between demographic variables and “OTOC” and “MAIRS-MS” scales: Independent sample t test was used for comparisons made according to gender variable; Anova test was used for data providing variance homogeneity in comparisons made with multiple group variables. In variables with 3 or more groups, it was determined in which group the difference was with advanced test (post-hoc). The reliability of the scales was tested with Cronbach Alpha coefficient. Pearson correlation coefficient was used to determine the correlation between MAIRS-MS and OTOC. The conceptual model presented within the scope of the research was created using SEM. The correlation between the scales and their effects on each other were modeled. The fit values of the model were evaluated. The analyzes were carried out with 5% error and 95% confidence interval (*p* < 0.05). *P* < 0.05 was used as statistical significance.

### Research hypothesis and questions

On the basis of the literature, the following hypothesis was generated:

- H1: There is a significant relationship between healthcare workers' readiness for medical AI and their perceptions of organizational openness to change.

In line with this hypothesis, the following research questions were sought to be answered;

- Research Questions 1: Healthcare workers are preparedness for the integration of medical AI within healthcare institutions.
- Research Questions 2: Healthcare workers are readiness for organizational change within healthcare institutions.
- Research Questions 3: Healthcare workers' readiness for medical AI differs according to demographic variables.
- Research Questions 4: Healthcare workers’' perception of openness to organizational change differs according to demographic variables.

## Results

### Demographic characteristics of health workers

Demographic characteristics of the participants are presented in Table 1. A situation assessment was made by determining the status and duration of AI usage of healthcare workers with descriptive information including general information about AI.

**Table 1:** Demographic characteristics of the participants

**Demografic Variable**		**F**	**%**
Gender	Female	123	63.1
	Male	72	36.9
Age	18–35 age	45	23.1
	36–45 age	48	24.6
	46–50 age	34	17.4
	51–54 age	34	17.4
	55–60 age	22	11.3
	61 and above age	12	6.2
Educational Status	High School	9	4.6
	Undergraduate	79	40.5
	Postgraduate and doctorate	62	31.8
	Medical Specialization	45	23.1
Position-Title	Physician	54	27.7
	Nurse	50	25.6
	Health Tech	38	19.5
	Other Health Worker	53	27.2
Working Time in the Profession	0–5 year	15	7.7
	6–10 year	26	13.3
	11–15 year	38	19.5
	16 and above	116	59.5
Working Time in the İnstitution	0–5 year	33	16.9
	6–10 year	24	12.3
	11–15 year	32	16.4
	16 and above	106	54.4
Department of science	Surgical Sciences	33	16.9
	Internal Medicine	40	20.5
	Basic Medical Sciences	23	11.8
	Other	110	56.4
AI usage status	Yes	110	56.4
	No	85	43.6
How many years have you been using the AI?	Neutral stance	32	16.4
	1–5 year	96	49.2
	6–10 year	11	5.6
	11–15 year	12	6.2
	16 and above	9	4.6
Total		195	100

### Data analysis

First, the data was organized by examining the extreme values and outliers. Extreme values were removed from the study, and the value of 3 was assigned to missing values using the value. Tabachnick and Fidel [48] argue that 5% missing data does not cause a problem. As a result of examining the research data, since the missing data was less than 5% and the missing data values of the scales were not found to be significant (*p* > 0.05), it was determined that the missing data occurred randomly and a value assignment method was used. As an application of assigning values instead of missing data, the close median value (3) was determined as a method compatible with the data set [49].

#### MAIRS-MS scale statistical analysis

The surface validity application of the scale was carried out with 10 healthcare workers and 3 expert healthcare managers. In order to test the understandability of the scale expressions in terms of meaning and content, face-to-face discussions were carried out. It was evaluated that the scale was understandable and suitable for the sample group and applied to the sample group and the scale factor structure was determined again by applying Exploratory Factor Analysis (EFA) in the analysis phase and confirmed with Confirmatory Factor Analysis (CFA).

The structure of the scale was tested by performing EFA and CFA. The obtained data were determined as KMO = 0.892 and Bartlett's = 0.000. (Chi-Square: 2471.012; df: 231; *p* = 0,000). In the EFA analysis, the principle component method and varimax rotation were used, and a 4-factor structure with an eigenvalue greater than 1 emerged. As a result of the EFA, in the healthcare workers group, which was a different sample group from the original scale, the following statements were included in the vision factor: “I can organize workflows in accordance with the logic of AI." (7), “I can express the importance of data collection, analysis, evaluation and safety; for the development of AI in healthcare"(8),” I can explain how AI applications in healthcare offer a solution to which problem." (13). Osborne and Fitzpatrick [50] stated that the EFA analysis produced factors that were only suitable for the presented data set in terms of the relationships between the items, and that these factors were rarely found in practice and had very low repeatability except under perfect conditions. Uyumaz et al. [51] stated that there was no measure of the generalizability of the analysis or the accuracy of the factor structure in their study, and therefore the researcher could reach different factors. The factor structure of the scale explains 62.50% of the total variance. The variances explained by the factors are 41.17%, 9.91%, 6.06% and 5.35%. As a result of the research, Skewness: −0.484 ± 0.174; Kurtosis: −0.067 ± 0.346 were found in the normality tests of the MAIRS-MS Scale. When evaluated together with the histogram and normal distribution graphs, it was concluded that the scale data showed normal distribution. There is no problem in using the scale in parametric tests. The mean of the MAIRS-MS Scale was found to be 3.40 ± 0.037. In the reliability analysis of the scale, Cronbach's Alpha value was found to be 0.928. Data related to the MAIRS-MS Scale are presented in Table 2.

**Table 2:** MAIRS-MS scale item analysis

İtems	Analysis N	Extraction	Mean ± SS	Cognition	Ability	Vision	Ethical	Corrected item-total correlation	Squared multiple correlation	Cronbach's Alpha if item deleted
1	195	0.617	3.33 ±.756	0.749				0.421	0.497	0.927
2	195	0.476	3.54 ±.851	0.498	0.464			0.525	0.430	0.925
3	195	0.649	2.91 ±.996	0.730		0.335		0.449	0.538	0.927
4	195	0.620	2.93 ± 1.005	0.678		0.395		0.522	0.572	0.926
5	195	0.613	3.21 ±.896	0.541	0.345	0.438		0.684	0.628	0.922
6	195	0.615	3.07 ±.859	0.541		0.537		0.632	0.622	0.923
7	195	0.683	3.18 ±.876	0.314		0.603	0.432	0.705	0.663	0.922
8	195	0.666	3.35 ±.925	0.325		0.671		0.687	0.612	0.922
9	195	0.562	3.66 ±.731	0.386	0.600			0.638	0.547	0.924
10	195	0.611	3.49 ±.833	0.381	0.542		0.308	0.714	0.657	0.922
11	195	0.769	3.67 ±.737		0.787			0.698	0.734	0.923
12	195	0.732	3.69 ±.766		0.752	0.309		0.699	0.718	0.922
13	195	0.650	3.41 ±.715		0.387	0.640		0.711	0.661	0.922
14	195	0.614	3.99 ±.739		0.755			0.483	0.460	0.926
15	195	0.466	3.53 ±.741		0.613			0.538	0.407	0.925
16	195	0.500	3.47 ±.808		0.591			0.589	0.567	0.924
17	195	0.706	3.21 ±.902			0.789		0.579	0.649	0.924
18	195	0.584	3.28 ±.860	0.320		0.637		0.649	0.592	0.923
19	195	0.514	3.44 ±.806			0.646		0.556	0.528	0.925
20	195	0.647	3.63 ±.847		0.536		0.573	0.597	0.660	0.924
21	195	0.750	3.70 ±.888				0.814	0.416	0.576	0.928
22	195	0.706	3.23 ±.903			0.311	0.773	0.462	0.502	0.927

Extraction Method: Principal Component Analysis. Rotation Method: Varimax with Kaiser Normalization

**Fig. 1:**
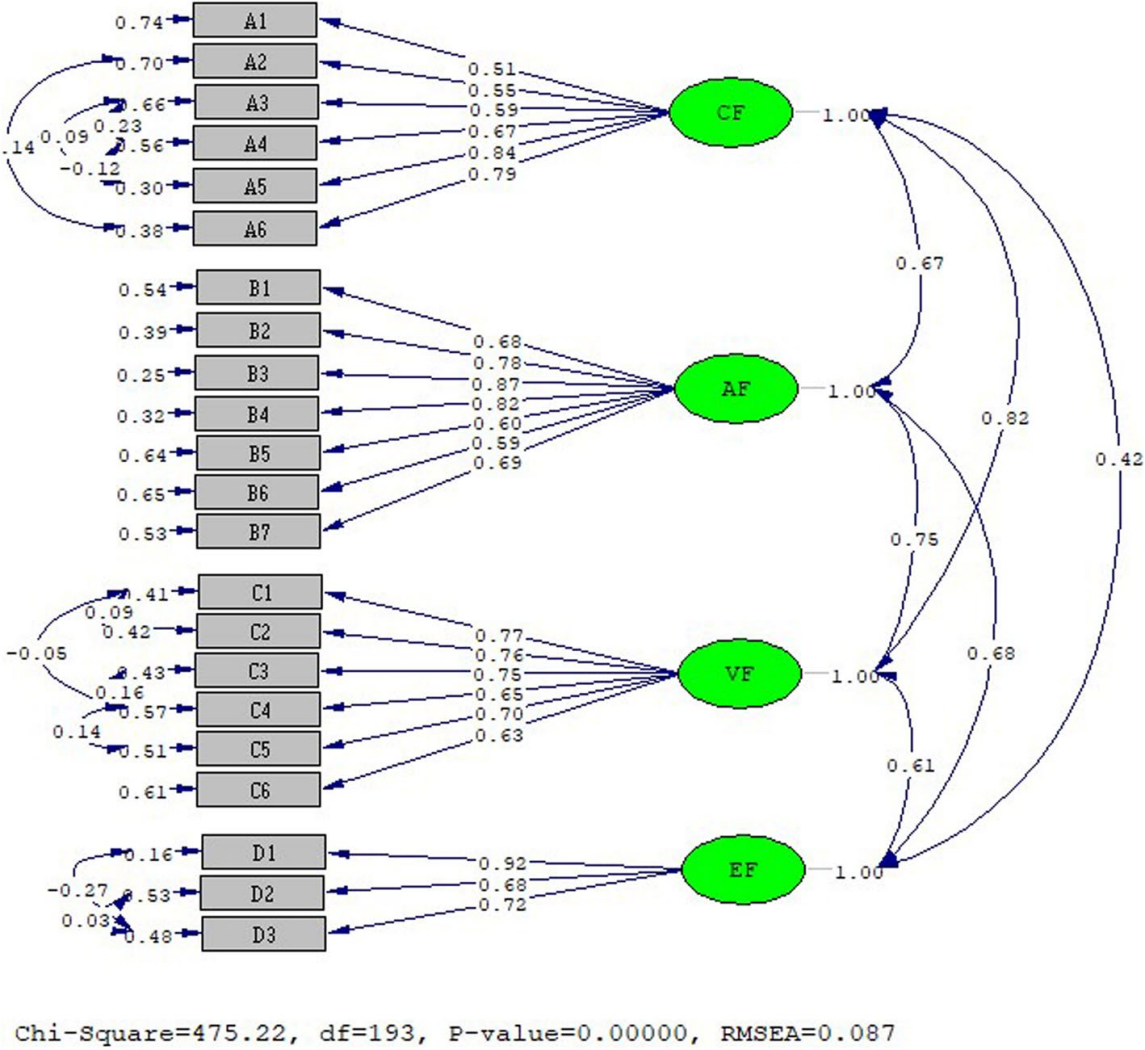
MAIRS-MS Scale CFA result standardized solution graph. (CF: Cognition factor; AF: Ability factor, VF: Vision factor; EF: Ethical factor)

In the study, CFA analysis was applied to the MAIRS-MS Scale using the Lisrel Estimate 8.8 program and structural validity was tested (Fig. 1).

As a result of examining, the CFA analysis fit values, acceptable values were reached and the construct validity of the scale was ensured. Fit values of the scale: RMSEA: 0.087 (0.05 ≤ RMSEA ≤ 0.1); Chi-square/SD (CMIN/DF): 2.46 (0 ≤ χ 2/sd ≤ 5); CFI: 0.96(0.90 ≤ CFI ≤ 0.95); GFI: 0.82 (0.90 ≤ CFI ≤ 0.95); RMR: 0.047 (0.05 ≤ SRMR ≤ 0.08); sRMR:0.066, NFI: 0.93 (0.90 ≤ NFI ≤ 0.95); IFI 0.96 (0.90 ≤ IFI ≤ 0.95); RFI) = 0.92 was determined [46, 48, 52].

OTOC Scale statistical analysis: The structure was tested by performing EFA and CFA analyses on the scale. The obtained data were determined as KMO = 0.850 and Bartlett's = 0.000 (Chi-Square: 762.941; df: 15; p = 0.000). In the EFA analysis, the principle component method and varimax rotation were used to obtain a single factor structure with an eigenvalue greater than 1. The resulting structure explains 68.49% of the total variance. The scale's Skewness = 276 ± 0.174; Kurtosis = −0.201 ± 0.346 was found. When evaluated together with histogram and normal distribution graphs, it was concluded that the scale data showed normal distribution. There is no problem in using the scale in parametric tests. In the reliability analysis of the scale, Cronbach's Alpha value was determined as 0.908. The OTOC Scale average was 3.95 ± 0.643. Data regarding the OTOC Scale are presented in Table 3.

**Table 3:** OTOC scale item analysis

İtems	Analysis N	Extraction	Mean ± SS	FACTOR 1	Corrected item-total correlation	Squared multiple correlation	Cronbach's alpha if item deleted
1	195	0.543	4.22 ±.716	0.737	0.636	0.485	0.906
2	195	0.670	4.00 ±.773	0.819	0.733	0.623	0.893
3	195	0.759	3.88 ±.794	0.871	0.802	0.681	0.883
4	195	0.674	3.75 ±.781	0.821	0.736	0.617	0.893
5	195	0.732	3.96 ±.792	0.856	0.781	0.668	0.886
6	195	0.730	3.91 ±.804	0.855	0.779	0.690	0.886

**Fig. 2:**
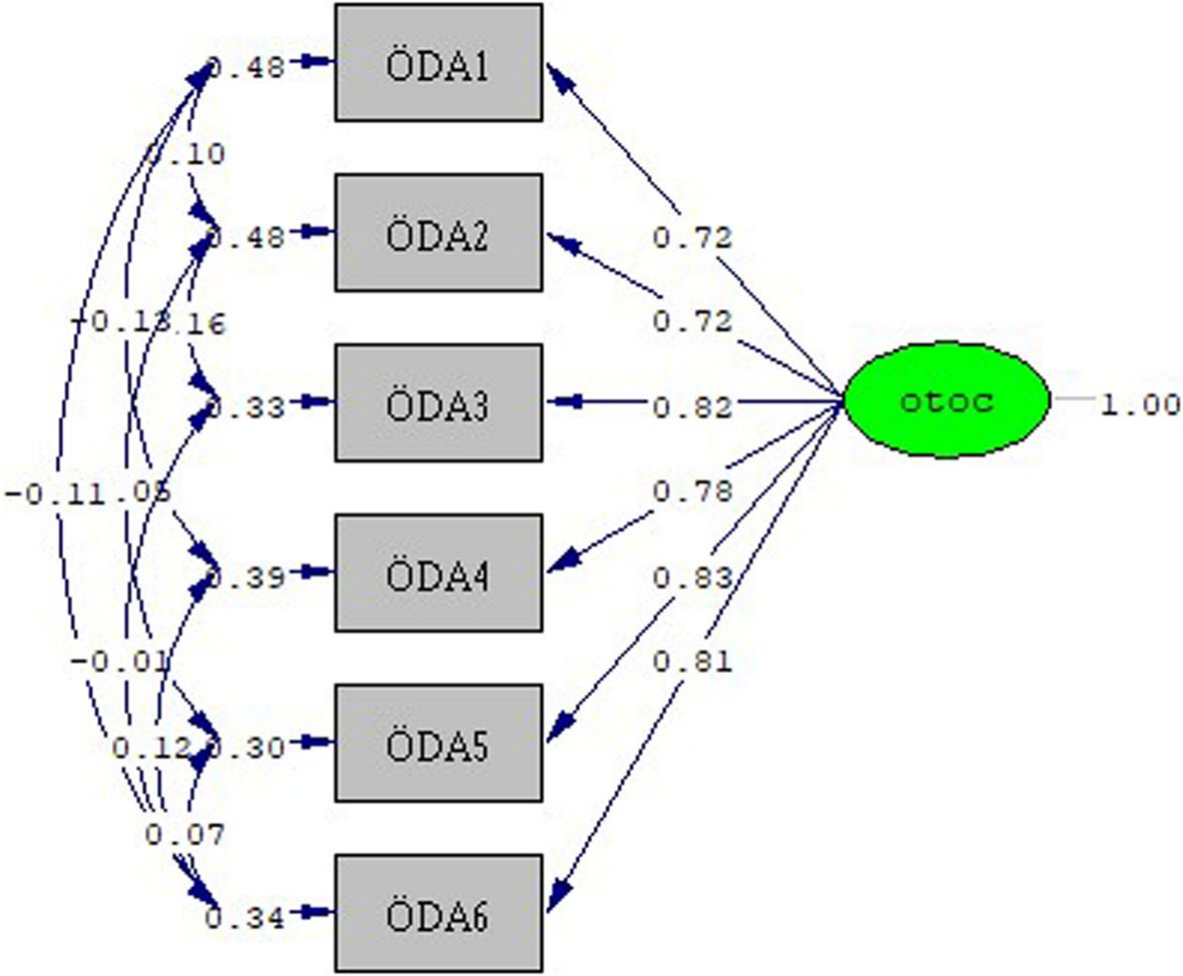
OTOC scale CFA result standardized solution graph

Using Listrel 8.8 Estimate program, CFA analysis was applied to the scale and its construct validity was tested (Fig. 2). As a result of examining the fit values of the CFA analysis, perfect fit values were reached and the construct validity of the scale was ensured. Fit values of the scale: RMSEA: 0.00; Chi-square/SD (CMIN/DF): 0.81; RFI: 0.99; CFI: 1.00; GFI: 1.00; RMR: 0.0042; sRMR: 0.0071, NFI: 1.00; IFI: 1.0. Validity and reliability studies of the scales were repeated and tested with the data obtained from the participants of the study. According to these results, comparison tests and hypotheses testing phase were started.

#### Correlation analysis of MAIRS-MS and OTOC scales and testing of hypotheses

This research was developed to determine the readiness of healthcare workers in healthcare institutions for medical AI and to explain the positive impact of change and its results in organizations through the phenomenon of openness to change. Correlation analysis was conducted to reveal the relationship between healthcare workers’ perception of readiness for AI and their perception of openness toward organizational change. Pearson correlation coefficient was used. In order to use Pearson correlation coefficient, equal interval scale should be used and data should be normally distributed. Our research meets these criteria. Correlation results are presented in Table 4.

**Table 4:** MAIRS-MS and OTOC scales correlation results

**Correlations**							
**Scales**		**OTOC**	**MAIRS-MS**	**Cognition**	**Ability**	**Vision**	**Ethical**
OTOC	Pearson Correlation	1					
	p						
MAIRS-MS	Pearson Correlation	0.236	1				
	p	0.001^**^					
Cognition	Pearson Correlation	0.140	0.814	1			
	p	0.050	0.000^**^				
Ability	Pearson Correlation	0.320	0.867	0.554	1		
	p	0.000^**^	0.000^**^	0.000^**^			
Vision	Pearson Correlation	0.100	0.882	0.668	0.647	1	
	p	0.165	0.000^**^	0.000^**^	0.000^**^		
Ethics	Pearson Correlation	0.232	0.664	0.313	0.591	0.488	1
	p	0.001^**^	0.000^**^	0.000^**^	0.000^**^	0.000^**^	

^**^Correlation is significant at the *p* < 0.001

**Fig. 3:**
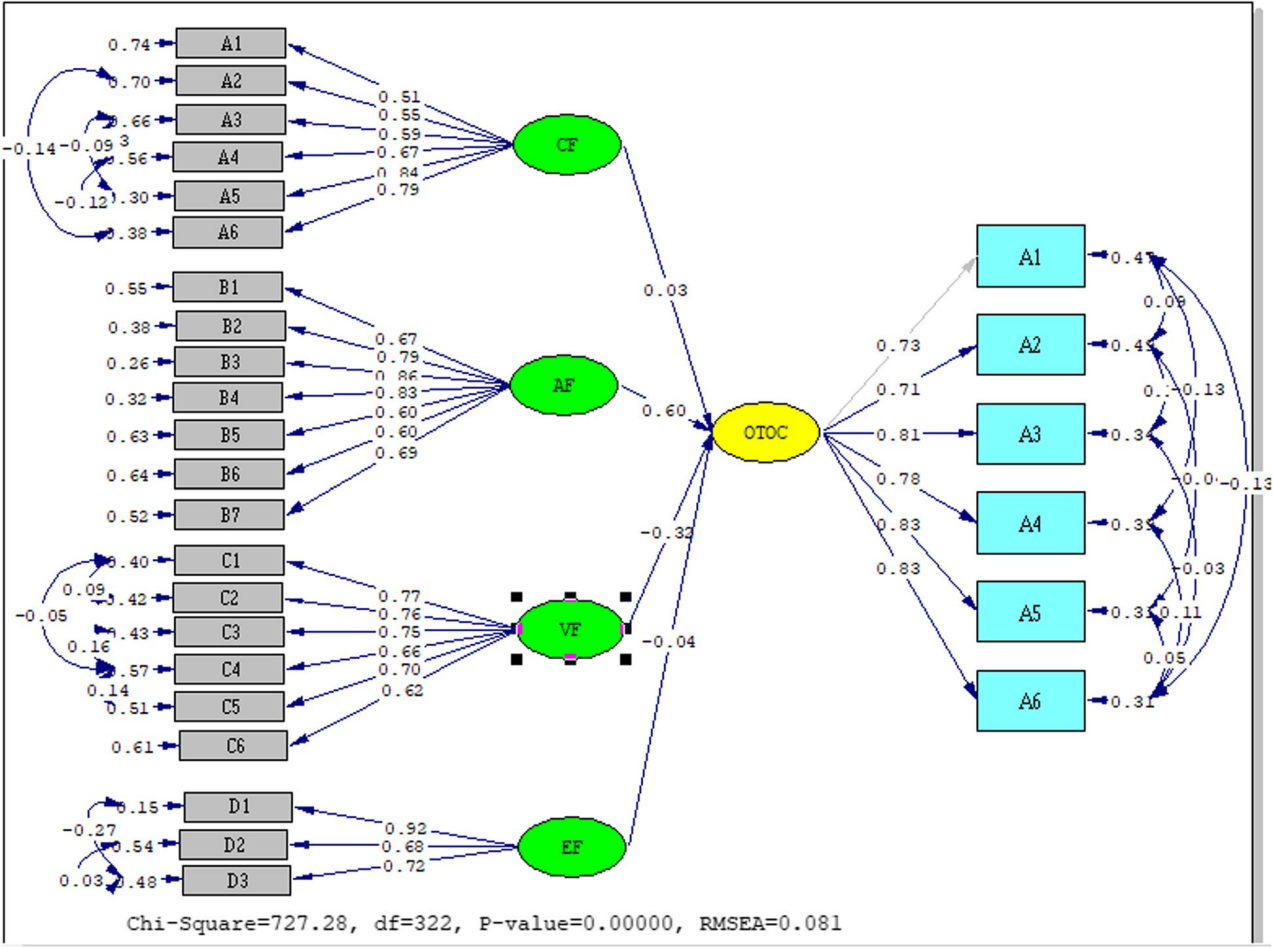
Modelling of the relationship between MAIRS-MS and OTOC. (CF: Cognition factor; AF: Ability factor, VF: Vision factor; EF: Ethics factor)

According to the research findings, a statistically significant low-level positive (*r* = 0.236; *R*^2^ = 5%) relationship was found between healthcare workers’ readiness for AI and their perception of openness toward organizational change (*p* < 0.05). Correlation significance values are used as; *r* < 0.20 −0 means no or very weak relationship, 0.20–0.39 (weak), 0.40–0.59 (medium), 0.60–0.79 (high), 0.80–1.0 (very high) relationship [53]. As a result of the research, healthcare workers’ readiness for AI and their perception of openness to organizational change were found to be weakly correlated and measured. Hypothesis H1 was accepted. (*r* = 0.236; *p* < 0.001). As healthcare workers’ AI readiness increases, their positive thoughts about openness toward organizational change also increase. A significant relationship was also found between healthcare workers’ perception of openness toward organizational change and the MAIRS-MS Scale sub-dimensions. A low-level positive significant relationship was found between openness to organizational change and the Ability factor (*r* = 0.320; *R*^2^ = 10%), and the ethics factor (*r* = 0.232; *R*^*2*^ = 5%) (*p* < 0.01). No relationship was found between the cognitive and vision factors (*p* > 0.01). In the study, healthcare workers’ AI readiness and perceptions of openness toward organizational change were modelled with SEM. Correlations between variables were also tested with the model (Fig. 3).

The SEM model was created using the lisrel program. The weak relationship between healthcare workers’ artificial intelligence readiness and their perception of openness to organizational change is also presented with a SEM diagram, and the fit values were determined as RMSEA: 0.081; Chi-square/SD (CMIN/DF): 2.42; RFI: 0.90; CFI: 0.95; GFI: 0.79; RMR: 0.050; sRMR: 0.075, NFI: 0.91; IFI: 0.95. The fit values indicate that the model provides acceptable fit. Correlation and covariance values between variables obtained as a result of SEM are presented in Table 5.

**Table 5:** MAIRS-MS and OTOC scales correlation results with SEM

	**Covariance matrix of latent variables**	**Mairs-Ms sub-dimension correlasion matrix**			
	**OTOC**	**Cognition**	**Ability**	**Vision**	**Ethical**
Correlation Matrix of Independent Variables					
Cognition	0.16	1.00			
Ability	0.38	0.67	1.00		
p		0.05			
t		13.63			
Vision	0.13	0.82	0.75	1.00	
p		0.04	0.04		
t		22.19	17.30		
Ethics	0.19	0.42	0.68	0.61	1.00
p		0.07	0.05	0.06	
t		6.31	12.58	10.29	

Note: *p* < 0.05

The correlation values obtained as a result of the Pearson correlation test were repeated with the SEM application and the proof was strengthened. Accordingly, when the relationship between the MAIRS-MS sub-factors and the Openness to Organizational Change Scale was examined; It can be said that there is a weak positive relationship between OTOC and ability and ethics factor.

#### Results of the comparison of scales according to demographic variables

As a result of the research, the OTOC scale average score was determined as 3.95 ± 0.643; the MAIRS-MS scale average score was determined as 3.40 ± 0.037.It is seen that the level of readiness of healthcare workers for AI and the perception of openness toward organizational change are above average. According to research questions 2 and 3, it was determined that healthcare workers are well prepared for the use of medical artificial intelligence and are open to organizational change. Comparison of demographic characteristics of participants and variables result present Table 6.

**Table 6:** Comparison of participants' demographic characteristics and variables

**Demographic characteristics**		**N**	**Percentage**	**MAIRS-MS**	**OTOC**	**MAIRS-Ms sub -dimensions**			
			**(%)**	**average. ± S.S**	**average ± S.S**	**Cognition**	**Ability**	**Vision**	**Ethical**
Gender	Female	123	63.1	3.33 ±.494	4.01 ±.622	3.06 ±.598	3.54 ±.548	3.25 ±.648	3.52 ±.594
	Male	72	36.9	3.52 ±.569	3.84 ±.667	3.33 ±.702	3.80 ±.615	3.40 ±.676	3.51 ±.905
	t-test			−2.507^t^	1.835^t^	−2.820^t^	−2.990^t^	−1.537^t^	0.017^t^
	p			0.013*	0.068	0.005*	0.003*	0.126	0.988
Age	18–35 Age	45	23.1	3.44 ±.513	4.02 ±.626	3.11 ±.706	3.79 ±.556	3.31 ±.628	3.56 ±.650
	36–45 Age	48	24.6	3.43 ±.596	3.92 ±.774	3.236 ±.668	3.66 ±.615	3.37 ±.758	3.41 ±.903
	46–50 Age	34	17.4	3.25 ±.559	4.04 ±.572	3.05 ±.590	3.43 ±.674	3.17 ±.665	3.41 ±.630
	51–54 Age	34	17.4	3.44 ±.482	3.83 ±.655	3.15 ±.587	3.64 ±.521	3.42 ±.666	3.57 ±.730
	55–60 Age	22	11.3	3.42 ±.398	3.91 ±.565	3.33 ±.534	3.57 ±.491	3.25 ±.366	3.59 ±.562
	61 Age above	12	6.2	3.38 ±.603	3.97 ±.425	3.04 ±.885	3.66 ±.552	3.20 ±.791	3.77 ±.671
	ANOVA			0.631^f^	0.537^f^	0.726^f^	1.529^f^	0.668^f^	0.768^f^
	p			0.676	0.748	0.605	0.183	0.648	0.574
Educational Status	Highschool^a^	9	4.6	3.24 ±.683	3.77 ±.186	3.20 ±.587	3.25 ±.816	3.18 ±.805	3.44 ±.971
	Bachelor Degree^b^	79	40.5	3.32 ±.421	3.83 ±.632	3.03 ±.582	3.54 ±.499	3.27 ±.552	3.46 ±.643
	Master ve Doctorate Degree^c^	62	31.8	3.40 ±.605	4.17 ±.709	3.22 ±.696	3.63 ±.657	3.30 ±.718	3.41 ±.723
	Medical Specialization^d^	45	23.1	3.57 ±.532	3.90 ±.555	3.29 ±.690	3.90 ±.476	3.40 ±.733	3.69 ±.793
	ANOVA			2.511^f^	3.797^f^	1.763^f^	5.491^f^	0.507^f^	1.173^f^
	p			0.060	0.011*	0.156	0.001*	0.678	0.321
	Post hoc Games howell				c > a(0.004) c > b(0.019)		d > b(0.001)		
Position-Title	Doctor^a^	54	27.7	3.57 ±.491	3.95 ±.564	3.33 ±.663	3.92 ±.461	3.39 ±.679	3.62 ±.767
	Nurse^b^	50	25.6	3.40 ±.580	4.28 ±.629	3.05 ±.731	3.67 ±.597	3.29 ±.765	3.68 ±.588
	Health technician^c^	38	19.5	3.29 ±.409	3.49 ±.582	3.09 ±.507	3.45 ±.480	3.27 ±.511	3.32 ±.592
	Other Employees^d^	53	27.2	3.30 ±.561	3.98 ±.586	3.13 ±.627	3.45 ±.645	3.26 ±.641	3.38 ±.828
	ANOVA			3.252^f^	12.868^f^	1.947^f^	8.095^f^	0.430^f^	2.880^f^
	p			0.023*	0.000*	0.123	0.000*	0.731	0.037*
	Post hoc Tukey			a > c.(0.047) a > d(0.037)	a > c(0.002) b > a(0.028) b > c(0.000) d > c(0.001)		a > c(0.001) a > d(0.000)		b > c(0.028)
Working time in the profession	0–5 year^a^	15	7.7	3.47 ±.435	3.73 ±.466	3.17 ±.649	3.85 ±.534	3.30 ±.441	3.51 ±.733
	6–10 year^b^	26	13.3	3.53 ±.380	4.25 ±.568	3.14 ±.460	3.87 ±.544	3.56 ±.464	3.43 ±.831
	11–15 year^c^	38	19.5	3.41 ±.629	3.91 ±.652	3.24 ±.762	3.61 ±.552	3.28 ±.765	3.53 ±.753
	16 and over year^d^	116	59.5	3.36 ±.534	3.92 ±.660	3.13 ±.652	3.56 ±.596	3.26 ±.679	3.53 ±.693
	ANOVA			0.844^f^	2.585^f^	0.273^f^	2.821^f^	1.512^f^	0.137^f^
	p			0.471	0.055	0.845	0.040	0.213	0.938
Working Time in the Institution	0–5 year^a^	33	16.9	3.49 ±.526	3.97 ±.564	3.16 ±.697	3.88 ±.576	3.35 ±.665	3.53 ±.600
	6–10 year^b^	24	12.3	3.49 ±.504	4.02 ±.688	3.29 ±.610	3.72 ±.440	3.50 ±.602	3.36 ± 1.062
	11–15 year^c^	32	16.4	3.31 ±.594	3.85 ±.708	3.14 ±.687	3.44 ±.709	3.23 ±.616	3.51 ±.555
	16 and over year^d^	106	54.4	3.38 ±.517	3.96 ±.640	3.13 ±.638	3.60 ±.554	3.27 ±.685	3.55 ±.714
	ANOVA			0.937^f^	0.335^f^	0.375^f^	3.507^f^	0.985^f^	0.465^f^
	p			0.424	0.800	0.771	0.016*	00.401	0.707
	Post hoc Tukey						a > c(0.012)		
Department	Surgery scienca^a^	33	16.9	3.44 ±.603	4.26 ±.649	3.22 ±.83818	3.78 ±.485	3.21 ±.825	3.54 ±.655
	Internal science^b^	40	20.5	3.63 ±.448	4.07 ±.622	3.26 ±.76692	3.95 ±.417	3.49 ±.641	3.92 ±.702
	Basic science^c^	23	11.8	3.40 ±.368	3.81 ±.494	3.08 ±.462	3.70 ±.512	3.36 ±.494	3.37 ±.683
	Other science^d^	99	50.8	3.29 ±.541	3.83 ±.643	3.11 ±.562	3.44 ±.621	3.25 ±.635	3.38 ±.707
	test			4.216^f^	4.806^f^	.652^f^	9.300^f^	1.595^f^	6.191^f^
	p			0.006*	0.003*	0.582	0.000*	0.192	0.000*
	Post-hoc Tukey			b > d (0.003)	a > c (0.047); a>d(0.004)		a > d (0.01) b > d (0.000)		b > c (0.015) b > d (0.00)

*S.S* Standard Deviation

^t^Independent sample ttest, ^f^ANOVA test, The comparison of variables resulting from the Anova test is shown with symbols a, b, c and d

*p < 0.05

There were found significant differences between health workers' readiness of Medical AI and demographic variables such as gender, position-title and department; however, no significant difference was found age, education, working time in the profession and institution.

Male health workers were found to have a high level of Medical AI readiness. (t-test: −2.507; *p* < 0.013). Doctors’ readiness of Medical AI was found to be higher than that of health technicians and the other group (F: 3.252; *p* < 0.023).İnternal medical science workers’ readiness of Medical AI were found to be higher for than other sciences workers (F: 4.216; *p* < 0.006).

There were significant differences between health workers’' perceptions of openness toward organizational change and demographic variables such as education, position-title and department; no significant difference was found gender, age, working time in the profession and institution.

Master's/doctorate degrees health workers' perception of openness to organizational change was higher than that of Highschool and Bachelor Degree graduates (F: 3.797 ± 0.011). Doctors' perception of openness to organizational change is higher than that of health technicians; nurses' perception of openness to organizational change is higher than that of physicians and health technicians; other health workers' perception of openness to organizational change is higher than that of health technicians (F: 12.868; *p* < 0.000).Surgical sciences health workers' perception of openness toward organizational change was higher than basic sciences and other sciences health workers. (F: 4.806; *p* < 0.003).

#### Comparison of the demographic characteristics of the participants and the sub-dimensions of the MAIRS-MS scale

Cognition factor: gender; Ability factor: gender, education, position-title, working time in the institution, department; Ethics factor: position-title and department were found to have significant differences. There was no difference between the Vision factor and demographic variables.

Male Health workers’ Medical AI readiness of cognition factor and ability factor level were found to be higher than that of female healthcare workers (t: −2.820; *p* < 0.005). (t: −2.990; *p* < 0.003). Medical specialization degree graduates Health workers’ Medical AI readiness of ability factor level was found to be higher than that of Bachelor Degree graduates (F: 5.491; *p* < 0.001). Doctors’ Medical AI readiness of ability factor level was found to be higher than health technologists and other health workers (F: 8.095; *p* < 0.000). Health workers’ Medical AI readiness of ability factor level of with 0–5 years of working time in the institution is higher than those with 11–15 years of working time in the institution (F: 3.507; *p* < 0.016).Health workers’ Medical AI readiness of ability factor level was found internal sciences and surgical sciences higher than those working in other sciences (F: 9.300; *p* < 0.000) Medical AI readiness ability factor level is the highest among surgical science health workers. Health workers’ Medical AI readiness of ethical factor level was found nurses higher than that of health technicians (F: 2.880; *p* < 0.037). Health workers’ Medical AI readiness of ethical factor level was found to be higher than that of the Internal Sciences department, basic sciences and other sciences departments (F:6.191; *P* < 0.000).

According to research questions 4 and 5, it was concluded that healthcare professionals' readiness for medical artificial intelligence and their perception of openness to organizational change differ according to demographic variables.

## Discussion

In the study, there was a statistically significant but low positive relationship between healthcare workers’ level of readiness for AI and their perceptions of organizational change. As the level of readiness for AI increases, their positive thoughts about organizational change also increase. Elkahlout [54] stated that the adoption of AI brings with it some difficulties (resistance to change, ethics, etc.) and that management should be careful in this regard. Fousiani et al. [55] stated that the use of AI and employee attitudes in organizations are affected not only by AI features but also by the climate of the organizations. And policy makers and senior management should empower employees [55]. Amin et al. stated that the readiness to adopt change is strongly linked to attitudes towards AI and that those who have a positive view of AI show less resistance [56]. Our research result is supported by the literature, but it is thought that the weak relationship between the variables is due to healthcare workers’ insufficient knowledge about AI-related change. It is thought that this should be supported by more detailed studies. Bisdas S.et al. [57] stated in their study with medical and dentistry students that they have a positive attitude towards AI and wants it to be included in their education. Schepman and Rodway [58] stated in their study that there is a negative attitude AI application in tasks such as medical treatment and psychological counselling and that there are ethical concerns. Oh et al. [59] stated in their study that most of the participants evaluated the use of AI in the medical field as beneficial. He considered the failure of AI to help in unexpected situations due to insufficient information as a possible problem. Çağlar [60] states that healthcare workers who are knowledgeable about the use of AI in the field of health and medicine have high levels of AI awareness.

In the research, it was determined that the level of readiness of healthcare workers for AI and their perception of openness toward organizational change in healthcare institutions differed according to demographic variables.

Significant differences were found between the AI readiness of healthcare workers and demographic variables such as gender, position-title and the department they work. Male employees, doctors and internal sciences employees have been found to be ready for AI. Çağlar [60] stated that the level of AI is higher in male health workers and doctors. Bisdas et al. [57] in his study, he stated that male employees and those who have curiosity of technology have a high perception of AI. In studies conducted with robots, it is stated that male employees have a positive opinion compared to women. The reason for this is shown as female employees highlighting risk and resistance factors, while male employees are quicker to accept [61]. In the study, female employees can be considered to be risky in the field of medical AI, while male employees are ready to use medical AI. Similarly, male healthcare workers were found to have high levels of cognitive and ability factors from the AI readiness sub-factors. It is thought that male healthcare workers are ready and able to systematically develop basic knowledge (cognitive). Younger and male participants show better knowledge and positive attitudes towards AI in healthcare [62]. Previous research conducted on undergraduate medical students in Germany has shown that men tend to trust the impact of technology and AI on radiology and medicine more [63]. This divergence in perceptions underscores the necessity for tailored strategies to address the distinct concerns and expectations of both groups to facilitate the effective integration of AI technologies in healthcare environments.

The study found that doctors' AI readiness level was high. Filiz et al., [9] found that physicians' anxiety of AI was lower than nurses. In the study by Ahmed et al. [64] 73.5% doctors stated that they were ready to apply AI practically in the future. The majority of doctors had basic knowledge about AI but did not have detailed knowledge about its applications in the medical field.

Al Fadeel et al. [65] in his study, there was no relationship between the perception of AI in health workers and the age of age, gender and working year. In our study, there was no relationship between age, education, working time in the profession and working institution and the level of AI. Filiz et al., [9] stated that the use of AI in health leads to moderate anxiety in health workers and that despite the difference between education and position; it did not determine a significant difference between age, gender, marital status and year of work. In the study of Filiz and Karagöz [66], there was no difference between the levels of AI anxiety of civilized status, age and gender. Ankara et al. [67] states that most of the health workers think that AI is useful in the field of health care. The integration of topics such as Metaverse, extended reality, blocks chain, etc. that emerged with AI into healthcare systems will shape the future of the healthcare system [68].

Cognition and ability factor perception of male health workers were found to be high. Specialist physicians in medicine have been found to have a high perception of ability factor of internal and surgical science employees, which have a working time in the institution for 0–5 years. Nurses and internal sciences employees have a high perception of ethical factor. Pakdemirli [69] has shown that health workers are moderately worried about AI practices in the field of health and that they do not have enough information about this. Chaieb et al. [70] states that health workers should be studied on AI practices. It is stated that the majority of the participants believe that AI is easy, that health professionals facilitate decision -making processes, increase productivity and accuracy and minimize costs. Shinners et al. [71] stated that health workers should be prepared urgently for the application of AI in the health sector.

There have been significant differences between the perception of openness of the healthcare workers toward organizational change and the education, position-title and the department in which they work from demographic variables. As a result of the research, groups with high perception of openness to organizational change; graduate and doctoral graduates, surgical science employees and physicians and nurses. Çinar and Toker [72] found that the operating room nurses are open to moderate change. Women, nurses aged 31–40 years, married and experienced in the group of 16 years and over, the management of the administration was found to be high. There was no significant difference between openness to organizational change and gender, age, working time in the profession and working time variables in the institution.

### Limitation

This study has some limitations. First, a convenience sampling method was used, which may limit the generalizability of the findings. On the other hand, the study was conducted in a single healthcare institution and therefore the generalizability of the results to all healthcare institutions and healthcare workers may not be guaranteed. It would be advantageous to replicate and expand this study using various samples to increase the applicability of the findings. Longitudinal studies are needed to support the generalizability of the findings. Further investigation of the effects on other settings (e.g. private or public) is needed. Second, the study was designed as a cross-sectional descriptive study and the cross-sectional design of the study only provides a snapshot of the relationship between the variables. The use of the convenience sampling method and the cross-sectional design pose significant threats to the validity and generalizability of the study. To address this limitation, future studies could expand the sample to include all healthcare institutions in Türkiye or even more diverse regions internationally to increase the external validity of the results. The study group consists of healthcare workers. The study is limited to healthcare workers. Since the time and cost elements of the study are taken into consideration, it is limited to healthcare workers (physicians, nurses, other healthcare workers) in different positions working in a university hospital in Istanbul. Participants were assured that their answers would remain anonymous and confidential. Nevertheless, it continues to be difficult to eliminate institutional concerns during the survey response process. The answers given to the survey in the study are considered correct. Data will not be collected from people other than healthcare workers and incomplete or incorrect information will not be included in the research. In the study, healthcare workers' perception of AI readiness and openness to change was evaluated with only two measurement tools in a certain time period. The increasing use of AI in the healthcare field in terms of time and technological developments reveals the necessity of supporting the findings obtained in the study by developing them with new studies.

## Conclusions

In the study, it was determined that healthcare workers are ready for the use of medical AI technology in healthcare institutions and perceive organizational change positively. This shows that changes to be made in the institution in light of developing technology will be adopted by healthcare workers. The results obtained show that using healthcare workers and the institution's AI applications in the healthcare field is a starting point for development, awareness and change and an important area of improvement from the perspective of managers and administrators. It can also support the development and well-being of healthcare workers by shaping the institution's healthcare policies and new applications. Future more detailed studies should investigate the benefits and risks of AI in healthcare institutions, compare the attitudes and behaviors of healthcare workers towards AI use and the use of AI in healthcare across regions and countries. Such comparative studies will contribute to the field of AI healthcare.

### Recommendation

- It is recommended that employees be made aware of the benefits of using AI in healthcare institutions and that necessary training activities be planned.
- It is necessary to question the openness of healthcare workers to organizational change and to be prepared for resistance to innovations to be implemented in institutions in the future.
- It is recommended that future studies investigate the perception, attitude and behavioral effects of AI use in healthcare workers in more depth and in larger sample groups. The importance of implementing comprehensive educational initiatives and other interventions to improve perceptions of AI and attitudes should be emphasized.

## Data Availability

The data sets used and/or analysed during the current study are available from the corresponding author.

## Abbreviations

AI: Artificial İntelligence
MAIRS-MS: Medical Artificial Intelligence Readiness Scale
OTOC: Openness toward Organizational Change Scale
SEM: Structural Equation Modeling
EFA: Exploratory Factor Analysis
CFA: Confirmative Factor Analysis
KMO: Kaiser-Meyer-Olkin
